# Disparities in surgical care for children across Brazil: Use of geospatial analysis

**DOI:** 10.1371/journal.pone.0220959

**Published:** 2019-08-20

**Authors:** João R. N. Vissoci, Cecilia T. Ong, Luciano de Andrade, Thiago Augusto Hernandes Rocha, Nubia Cristina da Silva, Dan Poenaru, Emily R. Smith, Henry E. Rice, on behalf of the Global Initiative for Children’s Surgery

**Affiliations:** 1 Duke Global Health Institute, Duke University Medical Center, Durham, NC, United States of America; 2 Division of Emergency Medicine, Duke University Medical Center, Durham, NC, United States of America; 3 Division of Global Neurosurgery and Neurology, Duke University Medical Center, Durham, NC, United States of America; 4 Graduate program in Health Sciences, State University of Maringá, Maringá, PR, Brazil; 5 Division of Pediatric Surgery, Duke University Medical Center, Durham, NC, United States of America; 6 Federal University of Minas Gerais, School of Economics, Center of Post-Graduate and Research in Administration, Belo Horizonte, Minas Gerais, Brazil; 7 Federal University of Minas Gerais, Faculty of Economics, Observatory of Human Resources in Health, Belo Horizonte, Minas Gerais, Brazil; 8 Division of Pediatric General and Thoracic Surgery, The Montreal Children's Hospital, McGill University Health Centre, Montreal, QC, Canada; 9 Robbins College of Health and Human Services, Baylor University, Waco, TX, United States of America; NPMS-HHC CIC / LSH&TM, UNITED KINGDOM

## Abstract

**Background:**

Health systems for surgical care for children in low- and middle-income countries remain poorly understood. Our goal was to characterize the delivery of surgical care for children across Brazil and to identify associations between surgical resources and childhood mortality.

**Methods:**

We performed a cross-sectional, ecological study to analyze surgical care for children in the public health system (*Sistema Único de Saúde*) across Brazil from 2010 to 2015. We collected data from several national databases, and used geospatial analysis (two-step floating catchment, Getis-Ord-Gi analysis, and geographically weighted regression) to explore relationships between infrastructure, workforce, access, procedure rate, under-5 mortality rate (U5MR), and perioperative mortality rate (POMR).

**Results:**

A total of 246,769 surgical procedures were performed in 6,007 first level/ district hospitals and 491 referral hospitals across Brazil over the study period. The surgical workforce is distributed unevenly across the country, with 0.13–0.26 pediatric surgeons per 100,000 children in the poorer North, Northeast and Midwest regions, and 0.6–0.68 pediatric surgeons per 100,000 children in the wealthier South and Southeast regions. Hospital infrastructure, procedure rate, and access to care is also unequally distributed across the country, with increased resources in the South and Southeast compared to the Northeast, North, and Midwest. The U5MR varies widely across the country, although procedure-specific POMR is consistent across regions. Increased access to care is associated with lower U5MR across Brazil, and access to surgical care differs by geographic region independent of socioeconomic status.

**Conclusions:**

There are wide disparities in surgical care for children across Brazil, with infrastructure, manpower, and resources distributed unevenly across the country. Access to surgical care is associated with improved U5MR independent of socioeconomic status. To address these disparities, policy should direct the allocation of surgical resources commensurate with local population needs.

## Introduction

Investment in all areas of childhood health, including surgical care, is critical to support functioning health systems in low- and middle-income countries (LMICs). Recently several initiatives have brought attention on surgical care in LMICs, including the Disease Control Priorities project,[1] the Lancet Commission on Global Surgery (LCoGS),[2] and the World Health Assembly resolution A68/15.[3] Despite these efforts, surgical care focused on the specific needs of children continues to be overlooked in the global health agenda.[4]

The Global Initiative for Children’s Surgery (GICS) is a consortium committed to improving the surgical care of children in LMICs.[5] GICS developed the Optimal Resources for Children’s Surgery (OReCS) program, which details guidelines for surgical care systems for children.(5) The OReCS program emphasizes that surgical care is best delivered through multiple tiers within national health systems, whereby the resources at different levels are commensurate with local population needs and the surgical complexity required.[5–7]

Brazil offers a rich environment to examine disparities in surgical care, as it has heterogeneous geography, health infrastructure, and socioeconomic resources across the country (GINI index 53.3 in 2017[8–10] Brazil has a large public health care system (*Sistema Único de Saúde*, *SUS)* and maintains several publicly available health system datasets.[8,11] Efforts to reduce health care disparities across the five Brazil regions (North, Northeast, Midwest, Southeast, and South have made great strides in recent years, particularly for primary care.[12] However, the delivery of surgical systems for children across Brazil remains poorly understood, leaving policymakers at a loss to improve the surgical care of children.

Geospatial analysis can help understand disparities in care in complex health systems, although the use of geospatial tools to examine surgical care for children in LMICs has been limited.[6,7] The goal of our study was to characterize the delivery of surgical care for children at a municipality level across Brazil, and to examine associations between geographic location, socioeconomic status, surgical delivery, manpower, infrastructure, and childhood mortality rates (under-5 mortality and perioperative mortality rates). Our secondary goal included testing the OReCS program for analysis of surgical care for children.

## Methods

### Study design

We conducted a cross-sectional, ecological study to analyze surgical care for children across Brazil within the public health system and to determine associations between surgical care and childhood mortality rates. We collected data on all children < 15 years of age undergoing a surgical procedure from 2010 to 2015 across Brazil using SUS datasets (DATASUS). Auxiliary data were collated from databases from the World Bank and the Brazilian Institute of Geography and Statistic (IBGE) (See Table 1 for all datasets and timeframes).[11] All data in DATASUS are de-identified, and are freely available through an open data access platform. We used geospatial analysis to explore relationships between surgical care infrastructure, workforce, utilization rates, and pediatric mortality rates. All health estimates were analyzed and summarized in line with the GATHER statement.[13]

**Table 1:** Data sources for analysis.

Source	Variables	Date Range	Data entries	Scope
DATASUS—Hospitalization information system (SIH)	• Hospitalization procedures performed • ICD code • Age of patient • Location of residence • Costs associate to the procedure • Hospital	2008–2015	267,248 procedures	Appendectomy (ICD 10 0DTJ4ZZ, 0DTJ0ZZ) Laparotomy (ICD 10 0WJP0ZZ) Hernia (ICD 10 0YQ54ZZ, 0YQ64ZZ, 0YQ50ZZ, 0YQ60ZZ, 0WQF4ZZ, 0WUF07Z, 0WUF0KZ, 0BQR4ZZ,0BQS4ZZ, 0BQR0ZZ, 0BQS0ZZ) Colostomy (ICD 10 0WQFXZ2) Abdominal wall reconstruction (ICD 10 0WQF0ZZ)
DATASUS—Mortality information system (SIM)	• Deaths of patients under 14 years old • The municipality of residence and of death • Mortality rate by municipality	2010–2015	326,459 deaths	All deaths between 2010 and 2015
CNES - National registration of health establishments	• Geolocation • Type of care provided • Accreditation	2014	6,498 hospitals	District and referral level hospitals
World Bank	• Gross national income (GNI) • Atlas index • GNI per capita • Income level classification	2010–2013	5565 municipalities	-
IBGE—Brazilian institute of geography and statistics	• Pediatric population by municipality • Gross domestic product (GDP) • GDP per capita	2008–2014	5565 municipalities	-

### Data inputs

#### Demographic and socioeconomic data

We extracted demographic and socioeconomic indicators from Brazilian Institute of Geography and Statistics (IBGE).[14] We used this data along with the Brazilian gross domestic product (GDP) to classify municipalities according to income groups as high income, upper-middle income, or lower-middle income as defined by the 2017 World Bank criteria of gross national income (GNI) per capita adjusted to US dollars (low income: GNI per capita $1,005 or less; lower middle-income: GNI per capita between $1,006 and $3,955; upper middle-income: GNI per capita between $3,956 and $12,235; high-income > $12,235).[15]

#### Pediatric surgery health care facilities infrastructure and human resources

Data about health care infrastructure, human resources, and location were obtained from *Cadastro Nacional de Estabelecimentos de Saúde*/National Register of Health Facilities.[11] We classified each hospital based on the complexity of infrastructure and human resources using criteria from the WHO as well as the OReCS program.[5, 16] For the purpose of this study, we classified facilities as community centers (defined as not capable of general anesthesia), first-level/district hospitals (defined as having a general surgeon and/or capable of general anesthesia), or referral hospitals (defined as having a pediatric surgeon and/or a pediatric intensive care unit). These levels are analogous to criteria used by the Brazilian health system, which categorizes centers as small hospitals or high-complexity centers (HCCs), with HCCs defined as capable of performing surgical procedures and/or obstetrical deliveries.[17]

#### Pediatric surgery delivery

We used a proxy set of five pediatric general surgical procedures to assess the delivery of surgical care across the public health system. This proxy set was based on the GICS OReCS document, which specifies representative surgical procedures to assess surgical care across a national health system. These five procedures included appendectomy, colostomy, hernia repair, laparotomy, and abdominal wall reconstruction for gastroschisis, omphalocele, or other indication (Table 1). Children undergoing each of these procedures were identified in the DATASUS Hospitalization Information System database (SIH) using International Classification of Diseases (ICD)-10 procedure codes. We summarized the annual rates for performance of all surgical procedures, and georeferenced them to the municipality of residence.

#### Mortality rates

We summarized annual all-cause under-5 pediatric mortality rates (U5MR) at the regional and municipality level using data from the Brazilian Mortality Information System database (SIM), which collects data on all deaths by age, sex, cause, and residence.[11] We also stratified mortality by under 1 year of age, under-5 years, 5–9 years, and 10–14 years.

We also collected procedure-based perioperative mortality rate (POMR) from the DATASUS Hospitalization Information System database (SIH) using the procedure codes for the proxy set of general surgical procedures. The SIH defines perioperative mortality as any death occurring during procedure. We summarized this data at the regional level for comparison of perioperative mortality rates across the country.

### Data analysis

Demographic data were summarized using descriptive statistics, with rates of procedures expressed as means with standard deviations. Choropleth maps were used to depict the distribution of each variable. To analyze the association between pediatric surgical care and mortality, we performed three complimentary geospatial analyses as described below. All spatial analyses were performed using ArcMap 10.5 (ESRI, Redlands, CA).

#### First geospatial analysis: Accessibility index

To evaluate geographic accessibility to surgical care, we used the two-step floating catchment area (2SFCA) method.[18] This technique uses a two-step technique to create an accessibility index for surgical care within a geographical area weighted by the population in that same space. In the first step, a geographic radius of 120 km surrounding each care facility was mapped to define the pediatric population potentially covered within a maximum travel distance of two hours as recommended by LCoGS.[2] Within that radius, a capacity ratio was calculated with number of hospital beds available for the pediatric population at each facility. In the second step, the capacity ratio of all facilities within 120 km from each municipality’s geographical center were added to create an accessibility index for each municipality. We used the 2SFCA method to define two independent accessibility indices for each municipality, one for first-level hospitals and a second for referral hospitals.

#### Second geospatial analysis: Spatial clustering of pediatric surgical delivery and mortality

To detect association between the delivery of surgical care and childhood mortality, we performed Getis-Ord-Gi analysis.[19] This measure of spatial heterogeneity uses autocorrelation and hot spot techniques to assess associations within spatial random variables. For our study, we identified hot spots (visually depicted as red areas) depicting clusters of municipalities with adjacent municipalities with high values for rates of a surgical procedure rate or U5MR, and cold spots (blue areas) depicting clusters with an adjacent low values regarding each indicator. Yellow areas mark locations where no clustering was observed. Geographic mapping was used to identify the distribution of each indicator within a spatial area.

#### Third geospatial analysis: Geographically weighted regression models of association between geographical access to pediatric surgical care and mortality

To further explore associations between surgical care and childhood mortality rates, we created geographically weighted regression (GWR) models. These models use multivariate regression to evaluate associations between potential predictors of surgical care delivery (income, procedure rate, and accessibility index) and childhood mortality rate, taking into account any spatial dependency. Spatial dependency is observed when the outcome variable (mortality) is explained by its location within a given geospatial area. We built sequential multivariate regression models taking into account spatial variability across Brazil to demonstrate if relationships between the predictors and mortality vary according to geography.[20] This technique identifies spatial clusters and performs local regression models within these clusters, generating an estimate by each geographical unit of analysis (municipalities).[21] Using this tool, we identified patterns of association of each variable with geographical dependency. Spatial dependency was evaluated by comparison of ordinary least square with spatial regression models. Considering the high spatial dependency in the model, we used a GWR model to identify local associations and demonstrate any post hoc effect of the model regarding space.

The performance of the GWR model was evaluated based on the adjusted R^2^ indicators, Akaike's information criterion parameters (AICc) and Moran's I of the residues. Sensitivity analyses were conducted by: (a) excluding each predictor; (b) aggregating procedures in one composite variable; (c) stratifying outcome by age. The coefficients regarding each predictor were standardized by z-score, and the results plotted using choropleth maps. Negative z-scores defined an inverse association between each predictor and U5MR, and positive z-scores characterized a direct association. The spatial self-correlation and OLS model were processed using GeoDa software 1·10·0·8 (Spatial Analysis Laboratory, Urbana, IL), ^17^ and the GWR model by GWR 4·0.[22] Choropleth maps were created using QGIS 2·14 software.[23]

## Results

From 2010 to 2015, a total of 246,769 surgical procedures were performed in children in the public health system in Brazil, yielding a mean annual rate of 66 procedures per 100,000 children. The surgery infrastructure for children includes 6,007 first-level/district hospitals, 491 referral hospitals and 40,612 community facilities. As of 2015, the surgical workforce included 1,514 pediatric surgeons, 82,626 general surgeons, and 16,212 anesthesiologists (rates per 100,000 children of 0.4, 56.2 and 13.9, respectively).

### Income distribution and geographical accessibility to pediatric surgical care

**Fig 1:**
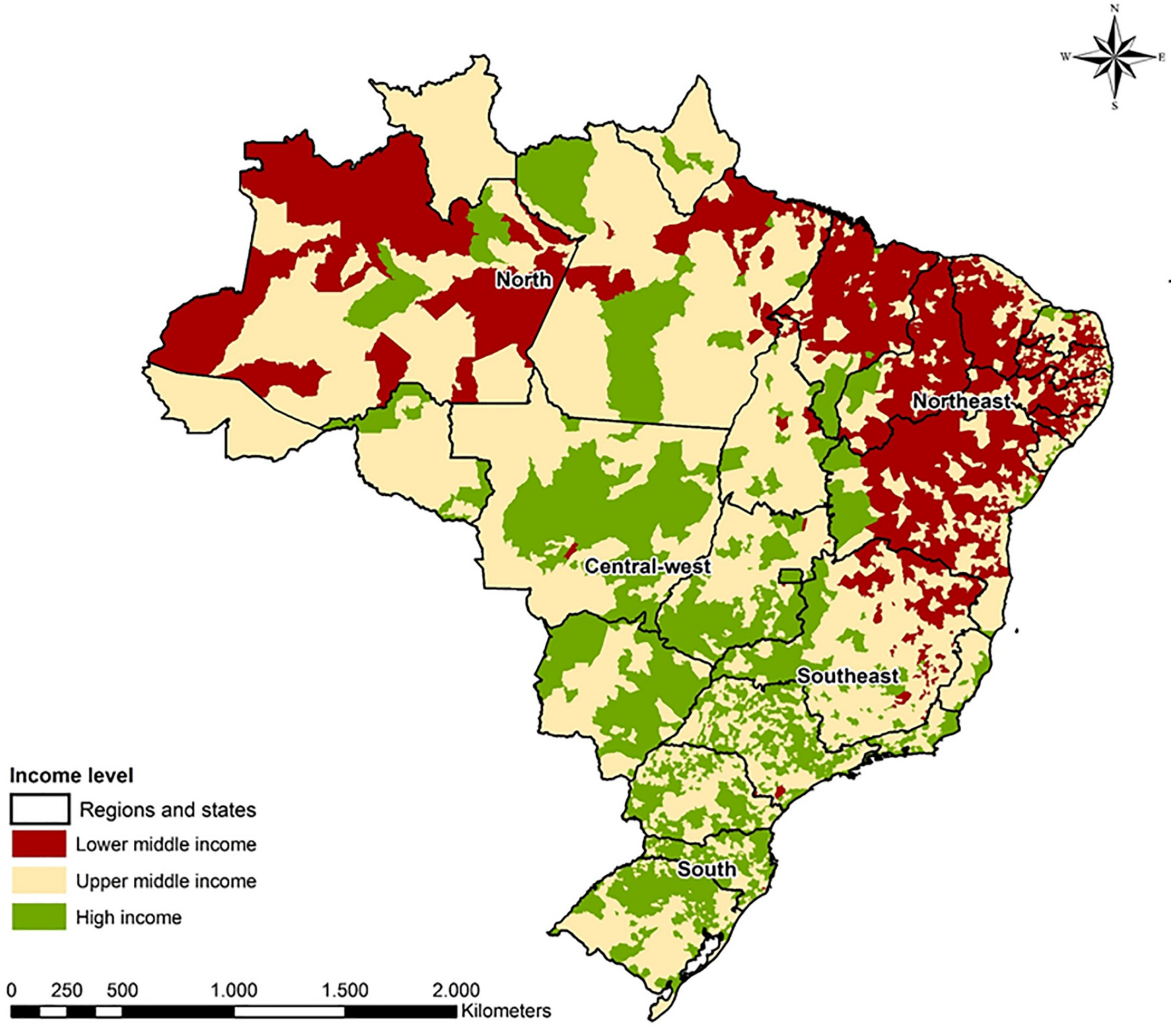
Income group distribution of Brazilian municipalities. Socioeconomic data were extracted from Brazilian Institute of Geography and Statistic (IBGE), and used with the Brazilian gross domestic product to classify municipalities according to income groups as defined by the World Bank as high income, upper-middle income, or lower-middle income. The map of Brazil was freely obtained in shapefile format (SHP) through online access to the website of the Brazilian Institute of Geography and Statistics (https://mapas.ibge.gov.br/bases-e-referenciais/bases-cartograficas/malhas-digitais.html).

We found wide disparities in income across Brazil with heterogeneous distribution of high income, upper-middle income, or lower-middle income municipalities as defined by the World Bank. In general, the wealthier areas were within South and Southeast regions, compared to poorer areas of the Northeast, North, and Midwest regions (Fig 1).

The pediatric surgical workforce is unevenly distributed across Brazil. There are 0.13–0.26 pediatric surgeons per 100,000 children in the North, Northeast and Midwest regions, with 0.6–0.68 pediatric surgeons per 100,000 children in the South and Southeast regions (Table 2). Similar heterogeneity in the health care professional workforce is seen for general surgeons, anesthesiologists, and emergency care providers (Table 2).

**Table 2:** Pediatric surgical procedures, workforce, hospital density, and pediatric mortality by region.

		N	Mid-west	Northeast	North	Southeast	South	Brazil
Procedure rates	Appendectomy	208,831	53.32 (44.58)	33.65 (26.95)	35.20 (32.38)	74.20 (51.58)	103.37 (71.67)	62.47 (55.78)
	Colostomy	4,359	0.12 (0.77)	0.15 (0.99)	0.14 (0.68)	0.17 (1.57)	0.12 (0.89)	0.14 (1.14)
	Hernia	6,483	0.51 (3.60)	0.19 (1.38)	0.69 (3.12)	0.54 (3.04)	0.53 (4.16)	0.43 (3.00)
	Laparotomy	23,248	2.81 (6.56)	2.88 (5.90)	2.96 (5.90)	2.22 (6.10)	3.38 (11.43)	2.79 (7.54)
	Abdominal wall	3,848	0.03 (0.24)	0.14 (1.04)	0.42 (2.18)	0.09 (0.92)	0.20 (1.32)	0.15 (1.17)
Manpower rate	Surgeons	82,626	57.22 (92.39)	22.51(50)	22.67 (44.91)	82.87 (158.46)	81.99 (143.48)	56.23 (120.04)
	Pediatric surgeons	1,514	0.26 (2.21)	0.13 (1.17)	0.15 (0.84)	0.68 (2.86)	0.6 (3.93)	0.41 (2.59)
	Emergency physicians	155,083	119.59 (149.76)	65.86 (99.74)	58.46 (98.21)	178.87 (260.75)	176.3 (265.85)	127.22 (210.3)
	Anesthesia	16,212	19.87 (44.42)	5.06 (16.57)	5.88 (17.57)	17.56 (39.07)	22.92 (64.3)	13.93 (40.91)
	Primary care	35,831	150.76 (117.07)	112.92 (70)	76.86 (63.48)	148.75 (164.61)	210.31 (244.93)	144.73 (160.01)
Infrastructure rate	District	6,007	18.28	56.16	14.78	51.84	26.1	167.16
	Referral	491	0.86	2.11	0.95	7.46	2.28	13.66
	Community facility	40,612	79.92	424.62	99.73	350.24	175.62	1130.13
Mortality rate	Less than 1 year	233,102	78.25 (39.22)	76.78 (26.82)	87.32 (43.55)	68.12 (29.57)	62.09 (31.84)	72.02 (32.35)
	Under-5	37,989	15.5 (21.79)	13.01 (9.45)	19.13 (15.01)	10.94 (11.42)	10.65 (12.52)	12.59 (12.84)
	5–9 years	22,815	9.46 (10.54)	8.41 (7.6)	9.93 (7.38)	7.31 (9.02)	7.15 (9.99)	8.02 (8.87)
	10–14 years	22,553	12.6 (13.05)	11.15 (8.24)	11.5 (8.4)	10.15 (10.32)	10.45 (12.23)	10.85 (10.3)
Perioperative mortality rate	All ages	349	0.17 (0.28)	0.17 (0.17)	0.11 (0.11)	0.14 (0.13)	0.16 (0.17)	0.15 (0.17)

All variables expressed as absolute number (N) nationally, as well as by mean rate (N/100,000 children) and standard deviation (σx) as stratified by region.

**Fig 2:**
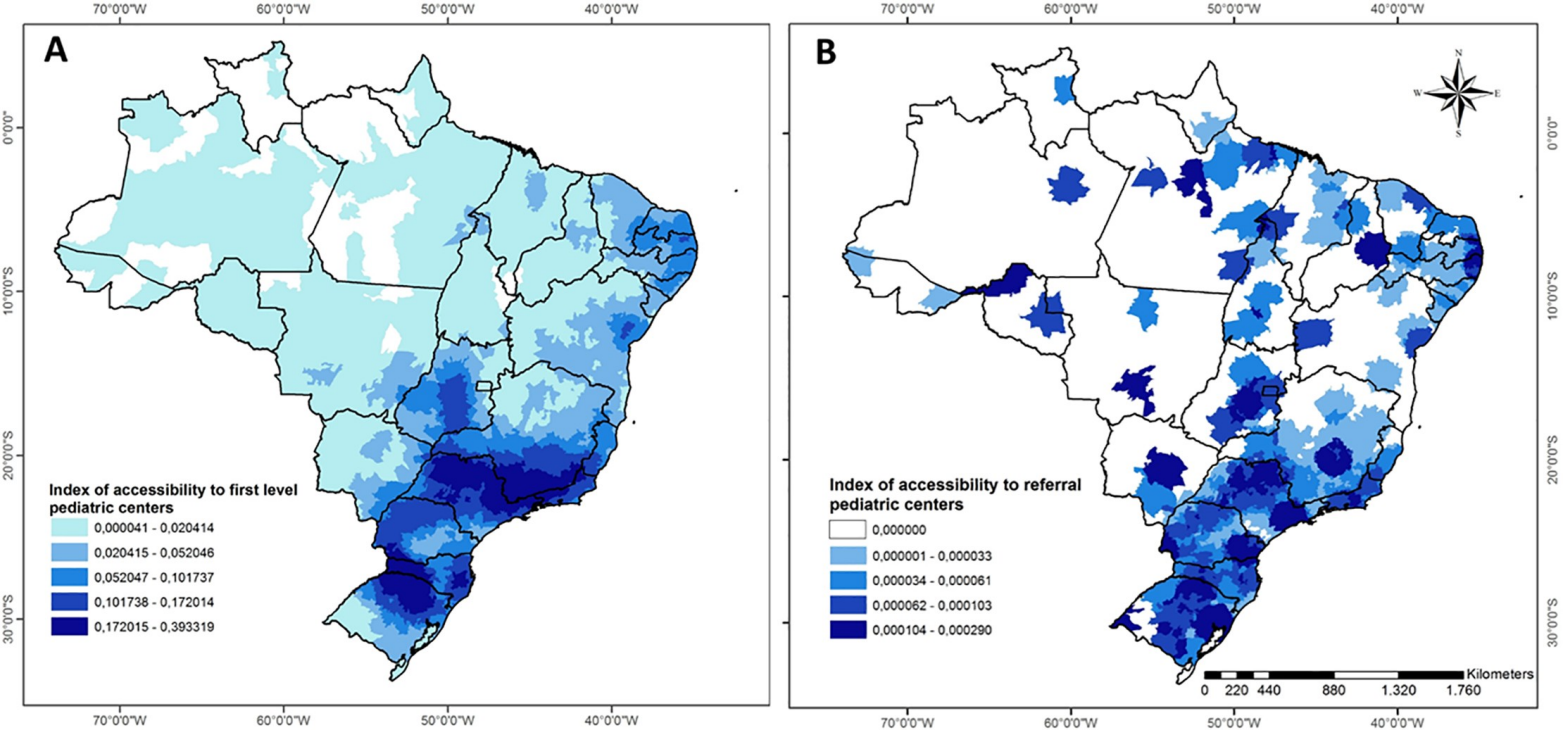
**Geospatial analysis of access to A) first level/district hospitals and B) referral-level hospitals for surgical care for children across Brazil using 2SFCA analysis.** Index values reflect the number of beds/pediatric inhabitant within 120 km radius from each municipality. For example, an index of 0·00041 means that in this municipality there are 0·00041 beds per pediatric inhabitant within a 120 km radius.

Access to surgical care is unequally distributed across Brazil, with higher rates of procedures and access to surgical care facilities in the South and Southeast compared to the Northeast, North, and Midwest (Fig 2). First-level/district hospitals were more accessible in the South and Southeast compared to the Northeast, North, and Midwest. The coastal region of the Northeast and some portions of the Midwest had higher access to first-level hospitals (Fig 2). Referral hospitals had lower access across the country compared to first-level/district hospitals. The Southeast and South regions had higher access for referral hospitals compared to other regions, although some areas in the Northeast and Midwest regions had similar access. Large portions of the Central-west, North, and rural Northeast regions had no access to any referral-level hospitals within a 120 km radius. These results indicate that approximately 364,732 children live beyond a 120 km range to first-level/district hospitals, and 6,586,168 children live beyond 120 km from any referral hospital.

### Spatial distribution and clustering of pediatric surgical delivery

**Fig 3:**
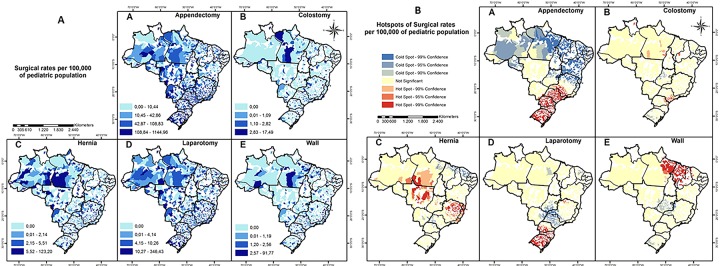
Geographic distribution across Brazil for rates of five proxy pediatric general surgical procedures (appendectomy, colostomy, hernia repair, laparotomy, abdominal wall defect). A) Rate of procedures/100,000 pediatric inhabitants performed at the municipality level. B) Hotspot and cold spots analysis demonstrates clustering of the rates of each procedure.

Of the five procedures used as a proxy set to assess surgical delivery, the most frequently performed procedure (by absolute number) was appendectomy (208,831), followed by laparotomy (23,248) and hernia repair (6,483) (Table 2). When weighted by population, procedure rates had wide geographic disparities, with some procedures not performed at all in many parts of the country (Fig 3). The rate of appendectomy and laparotomy was higher in more developed regions (South and Southeast) than in less-developed regions (North, Center-west, and Northeast). However, abdominal wall reconstruction had an opposite distribution, with a higher rate of procedures in the Northern regions than in the Southern regions (Fig 3A). The spatial correlation analysis highlighted hot spots for appendectomy and laparotomy in Southeast and South regions. The rate of colostomy showed a hot spot in Southeast region (Fig 3B).

### Surgical care and mortality rates

**Fig 4:**
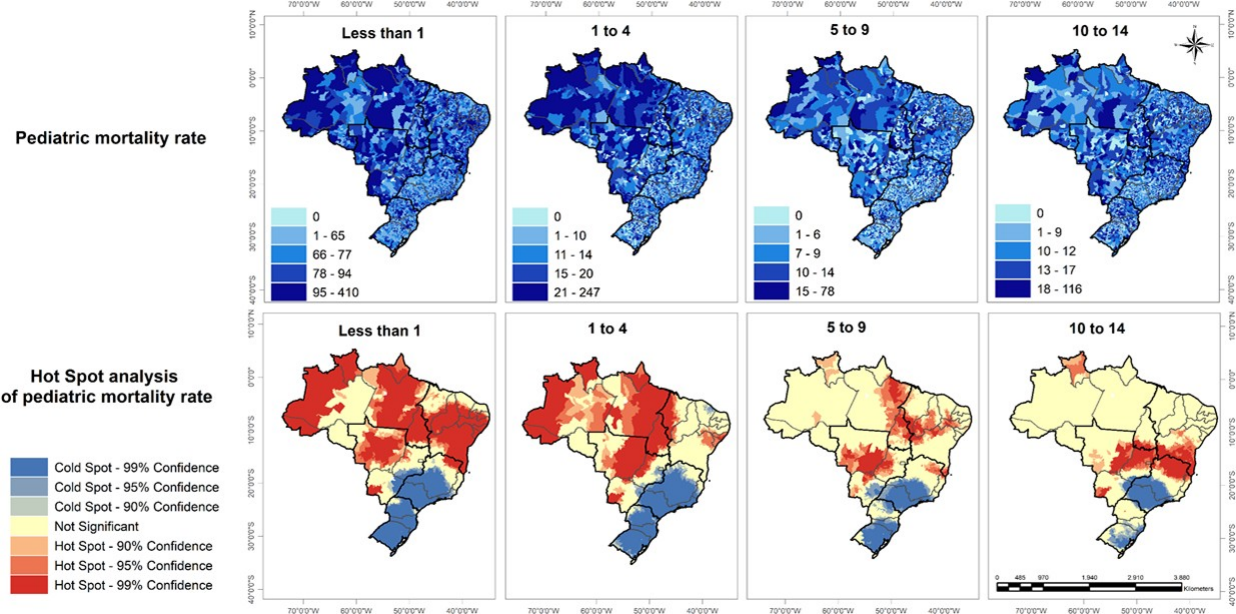
Geospatial distribution of pediatric mortality rate across Brazil. A) Distribution of pediatric mortality rates by age groups; and B) Hot spot (red) and cold spot (blue) of pediatric mortality rates.

Wide variations were seen in all-cause pediatric mortality rates across the country (Fig 4A). Spatial cluster analysis demonstrated high mortality rates for less than 1 year and under-5 mortality in most areas in the North, Northeast, and Midwest regions. Large portions of the Southeast and South demonstrated clustering of low mortality for all age groups (Fig 4B). In contrast to these disparities in all-cause pediatric mortality rates across Brazil, we found similar perioperative mortality rates across the five regions of Brazil for the proxy set of general surgical procedures (Table 2).

### Association between geographical access to surgery facilities, pediatric surgery delivery, and pediatric mortality rate

**Fig 5:**
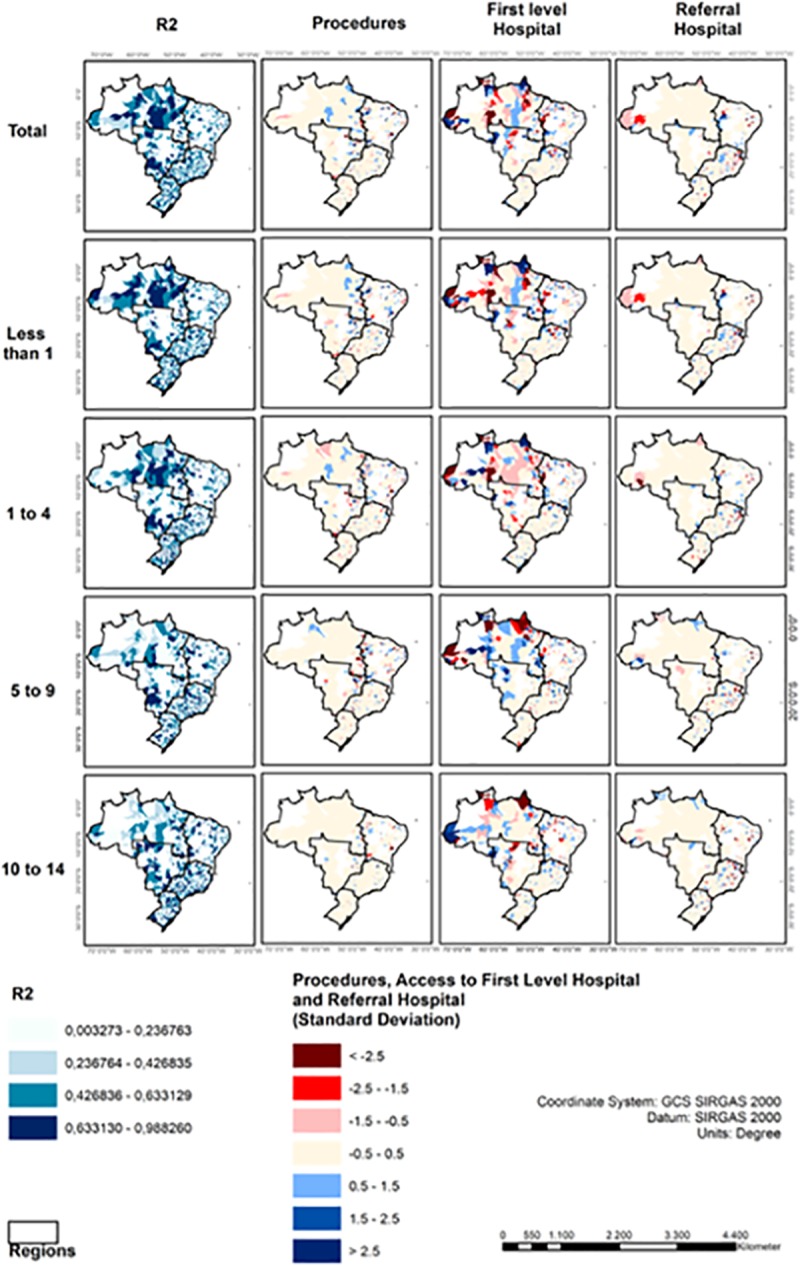
Geospatial representation of GWR model performance and association between variables (procedure rate, access to first/level or referral hospitals) and pediatric mortality by age group. Outcomes for income not represented, but were included in model analysis. Regions filled with darker colors represent areas with a higher adjusted R2, emphasizing better performance of the model. Blue areas are characterized by a positive association between the variable analyzed and pediatric mortality rate. Red areas depict an inverse (negative) association between the variable and the outcome.

Using GWR analysis, we found complex associations between each variable (access to care, surgical delivery, and income) and U5MR (Table 3 and Fig 5). In summary, the GWR analysis demonstrated: 1) Higher levels of surgery delivery were inversely associated with higher infant mortality rate and U5RM in the less developed areas of Northeast and North as well as silos across the country. Some sites in the South and Southeast showed a mixed pattern, with various associations between procedure rates and mortality. Increased access to first-level/district hospitals was associated with lower infant mortality rate and U5MR in the Amazonian area in the North and rural Northeast regions as well as Mid-west regions, although there were silos where access was not associated with mortality. In contrast, for older ages (5 to 14 years) there was not an association between access and mortality in the same areas; 3) Increased access to referral-level hospitals was associated with higher U5MR in the Northeast and Southeast regions, although the limited number of referral hospitals in restricted analysis of this association.

**Table 3:** Geographic weighted regression (GWR) models of association between pediatric mortality and access to pediatric surgical care.

	Overall deaths	Less than 1	1 to 4 yrs	5 to 9 yrs	10 to 14 yrs
Variables	z-score and SD	z-score and SD	z-score and SD	z-score and SD	z-score and SD
**Income**	-0.0213 / (0.8524)	-0.0134 / (0.852)	-0.0096 / (0.8518)	0.0010 / (0.8517)	-0.0267 / (0.8528)
**Procedures**	-0.0111 / (0.8519)	-0.0223 / (0.8525)	-0.005 / (0.8517)	0.0027 / (0.8517)	0.0127 / (0.8519)
**First level hospital**	0.0086 / (0.8518)	-0.0014 / (0.8517)	0.0128 / (0.8519)	0.0097 / (0.8518)	0.0132 / (0.8520)
**Referral-level hospital**	0.0038 / (0.8517)	0.0016 / (0.8517)	0.0042 / (0.8517)	-0.0016 / (0.8517)	0.0058 / (0.8517)
**AIC**	17365.142735	16897.119744	14616.799116	13634.968435	13919.43661
**Residuals Moran's I**	0.806587	0.445449988	0.127652	0.008524	0.22496
**Average local R2**	0.381109	0.387066116	0.376005	0.361774	0.399283

The performance of the GWR model was evaluated based on the adjusted R^2^ indicators, Akaike's information criterion parameters (AIC) and Moran's I of the residues

## Discussion

Brazil offers a valuable setting to examine surgical care across wide socioeconomic, demographic, geographic, and infrastructure ranges. The availability of large national datasets in Brazil supports detailed exploration of relationships between geographic location, infrastructure, workforce, socioeconomic status, and health outcomes. In our analysis, we found wide disparities in surgical care for children across Brazil, with facilities, manpower, and rates of surgical care distributed unevenly across the country. Geographic disparities in access to care are independent of socioeconomic status. Large portions of the population, particularly in poorer regions, live further than 120 km from any hospital capable of performing surgical procedures on children. In these areas, increased access to care is associated with decreased U5MR, a marker of the overall quality of health systems for children.

Our data aligns with recent analyses of surgical care for adults in Brazil, which showed wide disparities in manpower and surgical care delivery.[24, 25] The Brazilian government has long recognized challenges with disparities in health care delivery across the country, and have implemented several programs to increase health care access in rural areas, particularly for primary care services.[8, 12, 26] Our data suggests that there is a need to expand surgical care to minimize disparities in care delivery and improve health outcomes for children.

We noted different patterns of perioperative mortality rates and U5MRs across Brazil. The observed difference between these outcomes is likely largely related to the quality of the underlying data sources. The SIH, which is used for perioperative mortality rates, only records deaths occurring during the operative procedures themselves, and this dataset likely far underestimates the true POMR, which is generally accepted as within 30 days of surgery.[27] For this reason, we chose to use all-cause U5MR for our geospatial analysis, as this data is of far higher quality and thus allows for more accurate analysis of linkages between surgical delivery and childhood health. Future studies of surgical systems in Brazil may benefit from enhanced datasets to more accurately determine relationships between perioperative mortality and other perioperative outcomes, overall health outcomes, and delivery of surgical care.

Although the heterogeneity in surgical care across Brazil may be expected given the wide differences in socioeconomic resources across the country, our data confirm complex patterns of association between surgical access and socioeconomic status, and suggests that geographic disparities are independent of socioeconomic status. Most studies of health systems in LMICs examine care at a national level, and national statistics may not capture the local and regional variation that may exist in countries. Geospatial analysis facilitates examination of care at a municipality level, which is essential to understand delivery challenges at a local level. We found wide variations in procedure rates as well as access to surgical care at the municipality level across Brazil, even between neighboring municipalities, suggesting several policy considerations. For example, most areas of low appendectomy rates were in areas that had a low rate of complex cases, suggesting that hospitals in these municipalities may not have adequately trained manpower for high complexity cases. In other areas, low appendectomy rates were seen in hospitals that performed a high rate of more complex cases, suggesting that other cases may have taken precedence due to limited resources. These settings represent different capacity-building challenges; in some areas, additional hospitals and a general workforce may be necessary; in other areas, expansion of a specialized trained workforce may be required.

Geospatial analysis can help identify challenges high-quality care. As describe by in the Lancet Commission on High-Quality Health Systems, health systems should use locally relevant data to improve health care quality.[28] We found that in the Southeast region of Brazil, the complexity of care overall is well matched to hospital density and U5MR, suggesting that these hospitals have resources to provide care that is responsive to local needs, a marker of a high-quality health system. By contrast, in the North and Northeast, many areas have a high rate of complex cases such as laparotomy and abdominal wall reconstruction in regions of both low hospital density and high U5MR. These findings indicate that hospitals in the North and Northeast may be ill equipped to provide high-quality care. Care is provided by necessity in these regions despite being ill equipped for complex surgical care, as there may simply be no other local options. Additional studies of these challenges may require capture of surgery-specific measures of health care quality such as perioperative mortality rates, as well as other general measures of health care quality.[28, 29]

Our findings support the need to strategically address gaps in the surgical care for children within complex health systems as highlighted by the Global Initiative for Children’s Surgery (GICS).[5] The OReCS program emphasizes that surgical care for children is best delivered through multiple tiers within a comprehensive health system, whereby the resources at different levels are commensurate with local population needs and surgical complexity required.[5] Surgical care networks should be carefully organized within national systems, such that appropriate resources are provided for required surgical services within a geographic area.

There are several limitations to our study. First, DATASUS contains limited clinical information as well as perioperative outcomes. Second, have been raised about data quality in DATASUS,[11] and the use of ICD codes carries risks of coding errors.[30] Third, there are challenges with translating a 120 km distance from a care center to a 2 hour travel time across Brazil as recommended by the LCoGS,(2) as many regions in the Amazonia have limited access to roads. Fourth, some datasets in our analysis, such as perioperative mortality rate data from the SIH, include data from private sector. As private care forms up to 20% of health care in the South and Southeast regions,[31] use of information from these datasets may not be fully reflected through analysis of the public health sector. Finally, we recognize that the proxy set of procedures used to evaluate access to general surgical care does not reflect access to other pediatric surgical specialties.[31]

Despite these limitations, our analysis demonstrated wide geographic and socioeconomic disparities in delivery, infrastructure, workforce, and outcomes of surgical care children across Brazil. Limited access to surgical care is directly associated with increased U5MR. Importantly, geographic disparities in surgical care across Brazil are independent from socioeconomic status, suggesting that policies should focus on strategic allocation of surgical resources commensurate with local population needs. As well, future studies should address the costs, benefits, and economic metrics that guide expansion of surgical health systems. The OReCS program can help guide systems for surgical care, and can support local policy to improve the health care of children.
